# Supplementary material for: Incidence of respiratory distress and its predictors among neonates admitted to the neonatal intensive care unit, Black Lion Specialized Hospital, Addis Ababa, Ethiopia
Source: PLoS One. 2020 Jul 1;15(7):e0235544. doi: 10.1371/journal.pone.0235544 (PMC7329073; doi:10.1371/journal.pone.0235544)
Supplement: S2 Text — (DOCX) [file pone.0235544.s002.docx]

Table ***Incidence of respiratory distress and its predictors among neonates admitted to the neonatal intensive care unit, Black Lion Specialized Hospital, Addis Ababa, Ethiopia***

| Question  Number | Questions | Possible answers | |  |
| --- | --- | --- | --- | --- |
| Questions for the mother | | | | |
| Socio-demographic characteristics | | | | |
| 101. | Age |  | |  |
| 102. | Place of residence | 1. ruler  2.urban | |  |
| b. Obstetric and medical related factors | | | | |
| 1. | Number of Gravidity |  | |  |
| 2. | Number of Parity |  | |  |
| 3 | Does the mother have ANC follow up? | 1. Yes……  2. No…… | |  |
| 4 | Was the current pregnancy multiple(twin)? | 1.yes  2.no | |  |
| 5 | What was her Current mode of delivery? | 1.spontanous vaginal delivery  2.cesarean section  3. Instrumental | |  |
| 6 | Does the neonates breastfeed? | 1. yes  2.no | |  |
| 7 | If yes, when was the breast feed initiated | 1. <1 Hr  2. [1, 2] Hr  3. >2 Hr | |  |
| c. Mothers medical problem | | | | |
| 8 | Has she been diagnosed with any medical problems? | 1. Yes  2. No. | | To Q NO 2010 |
| 9 | If yes for question no 207, what was the diagnosis | 1. HIV  2. Hypertension  3. anemia  4.other…… | | |
| **II. Questions for the preterm neonate** | | | | |
| **a. Socio demographic / Identifications** | | | | |
| 1. | ID no- |  | |  |
| 2 | date of admission |  | |  |
| 3 | Age in day |  | |  |
| 4. | Sex | 1. Male  2. Female | |  |
| 5 | Gestational age at birth in weeks |  | |  |
| 6. | Weight in grams |  | |  |
| 7. | APGAR score of | 1.1^st^minute…  2.5^th^ minute…  3.10^th^minute… | |  |
| **b. Diagnosed comorbidities (medical problems)** | | | | |
| 9 | Had the neonate been diagnosed with any medical disorders? | a. Yes  , b. No | | To Q NO309 |
| 10 | If yes to question no 309, what was the diagnosis? | a. Respiratory distress  b. Jaundice  c. Perinatal Asphyxia  d. Hypothermia  E. Sepsis  F. Other(specify) | |  |
| 11 | Length of hospital stay |  |  | |
| 12 | Date of discharge |  | |  |
| 13 | Time of RD diagnosis |  | |  |
| 14 | Patient status | a. death  b. alive  c. referred  d. other… | |  |
