# Supplementary material for: Incidence of respiratory distress and its predictors among neonates admitted to the neonatal intensive care unit, Black Lion Specialized Hospital, Addis Ababa, Ethiopia
Source: PLoS One. 2020 Jul 1;15(7):e0235544. doi: 10.1371/journal.pone.0235544 (PMC7329073; doi:10.1371/journal.pone.0235544)
Supplement: Attachment — Submitted filename: response to reviewers for incidence of rd for plose one.docx [file pone.0235544.s004.docx]

**Dear Academic Editor!**

**PLOS ONE**

**Response to Reviewers**

I am pleased to resubmit for publication version of “**Incidence of respiratory distress and its predictors among neonates admitted at neonatal intensive care unit, Black Lion Specialized Hospital, Addis Ababa, Ethiopia**” for a review as original research in PLOS ONE.

The comments of the reviewers were highly insightful and enabled us to greatly improve the quality of our manuscript. Therefore, based on the reviewers’ concerns we have made extensive edition in our manuscript. In the following pages, we have addressed yours’ concerns in a point by point format.

We look forward to hearing from you at your earliest convenience.

Thank you for your consideration of this manuscript!

Sincerely,

**Yared asmare aynalem**

On behalf of authors

**Editor and Review Comments to the Authors**

**Editor comments**

Dear mr Aynalem,

Thank you for submitting the above manuscript to PLOS ONE. During our internal evaluation of the manuscript, we found significant text overlap between your submission and the following previously published works:

<https://www.uptodate.com/contents/overview-of-neonatal-respiratory-distress-disorders-of-transition>

<https://www.macpeds.com/documents/Respiratory%20distress%20in%20newborn%20PIR.pdf>

<https://www.longdom.org/abstract/prevalence-of-unwanted-pregnancy-and-associated-factors-among-women-in-reproductive-age-groups-at-selected-health-facili-41439.html>

<https://www.intechopen.com/books/respiratory-management-of-newborns/respiratory-distress-and-management-strategies-in-the-newborn>

We would like to make you aware that copying extracts from previous publications, especially outside the methods section, word-for-word is unacceptable. In addition, the reproduction of text from published reports has implications for the copyright that may apply to the publications.

Please revise the manuscript to rephrase the duplicated text, cite your sources, and provide details as to how the current manuscript advances on previous work. Please note that further consideration is dependent on the submission of a manuscript that addresses these concerns about the overlap in text with published work.

Response: thank you very much for your important comment. Based on your recommendations, we have made revision of the manuscript accordingly in all over the mascripit. We thank you very much for this important recommendation. We acknowledge that English is not our first language and we have edited the manuscript by a professional language editor (a fluent, native Australian, English-language speaker thoroughly edited the manuscript for language usage, spelling, and grammar) before submitting the revised version. The formatting of the text and document (text sizes and grammatical errors) were also edited. See the manuscript with track changes.

. Therefore, based on the editor’s and the reviewers’ concerns we have made extensive edition in our manuscript. Especially we have extensively edited the manuscript by a professional language editor (a fluent, native Australian, English-language speaker thoroughly edited the manuscript for language usage, spelling, and grammar) before submitting the revised version.also **An editor from excision editing member whose name is called dr Ryan bell (CEO and chief editor, excision editing), edits the whole manuscript.so that we think that we try to improve	the** grammar, typographical errors, and consistency.A professional	editor called professior Pammla Margaret Petrucka(College of Nursing, University of Saskatchewan) also gives a constractive feedback including the English-language edition.

The formatting of the text and document (text sizes and grammatical errors) were also edited. In the following pages, we have addressed yours’ concerns in a point by point format.

We have etisivly edited the title page ,we removed any abrivations in the abstract part.

The background parts of the manscripits was rephrasing intenssivly

we have correct and rewrite all the sentence in the methods,result ,discussion and reference part.for addtionannl we have attached the manscripit with its track changes

**Incidence of respiratory distress and its predictors among neonates admitted at neonatal intensive care unit, Black Lion Specialized Hospital, Addis Ababa, Ethiopia**

Yared Asmare^1^*, Hussien Mekonen^2^, Tadesse Yirga^3^, Tesfa Dejenie Habtewold^4^,Aklilu Endalamaw^5^ ,Wondimeneh Shibabaw^1^

^1^College of Health Science, Debre Birhan University, Debre Birhan, Ethiopia

^2^College of Health Sciences, School of Nursing and Midwifery, Addis Ababa University

^3^College of Health Science Debre Markos University, Debre Markos, Ethiopia

^1,3^Department of Epidemiology, University Medical Centre Groningen, University of Groningen, Groningen, the Netherlands

^5^ Department of Pediatrics and Child Health Nursing, College of Medicine and Health Sciences, Bahir Dar University, Bahir Dar, Ethiopia

Address

*Corresponding author: Yared Asmare (YA): [yaredasmare@yahoo.com](mailto:yaredasmare@yahoo.com)

Yared Asmare (YA): [yaredasmare@yahoo.com](mailto:yaredasmare@yahoo.com), P.O.Box 445, Debre Birhan, Ethiopia

Hussein Mekonen (HM): [hussen_mk@yahoo.com](mailto:hussen_mk@yahoo.com) P.O.Box 11240 Addis Ababa, Ethiopia

Tadesse Yirga (TY): [tadesseyirga680@gmail.com](mailto:tadesseyirga680@gmail.com)

Tesfa Dejenie (TD): [tesfadej2003@gmail.com](mailto:tesfadej2003@gmail.com)

Aklilu Endalamaw (AE): [yaklilu12@gmail.com](mailto:yaklilu12@gmail.com)

Wondimeneh Shibabaw (WS): [wshibabaw2015@gmail.com](mailto:wshibabaw2015@gmail.com)

**Abstract**

**Background:** Although respiratory distress is one of the major causes of neonatal morbidity and mortality throughout the globe. It is a serious concern more of in resource limited nations, like Ethiopia. Besides, few studies are available in developing countries. Data from different settings is needed to tackle it. Therefore, we intended to assess the incidence and predictors of respiratory distress among neonates who were admitted in Neonatal Intensive Care Unit at Black Lion Specialized Hospital, Ethiopia.

**Methods:** Institution-based retrospective follow-up study was conducted among 571 neonates from January 2013 to March 2018. Data were collected by reviewing patients’ charts using a systematic sampling technique with a pretested checklist; entered using Epi-data 4.2 and analyzed with STATA 14. Median time, Kaplan-Meier survival estimation curve, and log-rank test were computed. Bivariable and multivariable Gompertz parametric hazards models were fitted to detect the determinants of respiratory distress. Hazard ratio with a 95% confidence interval was calculated. Variables with reported p-values < 0.05 were considered statistically significant.

**Results**: The proportion of respiratory distress among of neonates admitted in Black Lion Specialized Hospital neonatal intensive care unit was 42.9% (95% CI: 39.3-46.1%) with an incidence of 8.1/100(95% CI: 7.3, 8.9).. Being male [AHR=2.4 (95% CI:1.1,3.1)], born via caesarean section [AHR:1,.9 (95%CI:1.6,2.3)], home delivery [AHR :2.9 (95%CI:1.5, 5,2)], maternal diabetes mellitus (AHR 2.3 (95% CI: 1.4,3.6)), preterm birth [AHR:2.9 (95%CI:1.6, 5.1)] and APGAR score less than 7 [AHR: 3.1 (95% CI:1.8,5.0)] were found to be significant predictors of respiratory distress in neonates.

**Conclusions:** In this study, the proportion of neonatal respiratory distress was high. Preterm birth, caesarean section delivery, APGAR score<7, sepsis, maternal diabetes mellitus and home delivery were significant predictors. All concerned bodies should work on preventing respiratory distress and give special attention to its multifactorial causes. The incidence of respiratory distress among neonates was found to be high. Those neonates delivered at home, delivered through caesarean section, preterm neonates, whose APGAR score<7, and born from diabetic mothers were more likely to develop respiratory distress. Thus, it is indicated to promote health institutional delivery. Besides, a need to establish and/or strengthen strategies to prevent the occurrence of respiratory distress among babies with low APGAR score, preterm babies, born from diabetes mellitus mothers, and delivered through caesarean section.

**Keywords:** Ethiopia, neonate, respiratory distress, NICU

**Background**

Respiratory distress (RD) is a common newborn problem immediately following birth. It is typically triggered by abnormal respiratory function during the transition from fetal to neonatal life. Tachypnea, intercostal retraction, nasal flaring, audible grunting, and cyanosis are the common manifestations of RD. The successful transition from fetal to neonatal life requires a series of rapid physiologic changes of the cardiorespiratory systems. These changes result in redirection of gas exchange from the placenta to the lungs and comprise replacement of alveolar fluid with air and onset of regular breathing [1]. Although RD may be transient, when it becomes persistent distress there is a need for proper diagnostic and therapeutic interventions to optimize outcomes and minimize morbidity.

RD is one of the most common reasons for neonates to be admitted to the neonatal intensive care unit (NICU) [2, 3]. Fifteen percent of term infants and 29% of late preterm infants admitted to the NICU develop significant respiratory morbidity ??source. This incidence is even higher for infants born before 34 weeks’ gestation [4] ([2](https://www.ncbi.nlm.nih.gov/pmc/articles/PMC4533247/#B2)).

Certain risk factors increase the likelihood of neonatal RD. Prematurity, low first and fifth minute APGAR scores, meconium-stained amniotic fluid (MSAF), caesarian section delivery, gestational diabetes, maternal chorioamnionitis, premature rupture of membranes [5] and oligohydramnios, as well as structural lung abnormalities are some predictors identified within previous studies [4, 6-9]. Other common causes also include transient tachypnea of the newborn, meconium aspiration syndrome, pneumonia, sepsis, pneumothorax, and persistent pulmonary hypertension of the newborn [10].

In contrast, the risk decreases with each advancing week of gestation and birth through spontaneous vaginal delivery [11].

Regardless of the cause, if not recognized and managed quickly, RD can escalate to respiratory failure and cardiopulmonary arrest and even to death. Therefore, it is imperative that any health care practitioner caring for newborn infants can readily recognize the signs and symptoms ofRD, differentiate various causes, and initiate management strategies to prevent significant complications or death. Consequently, neonates in need of critical medical attention are usually admitted to the NICU. These infants tend to be preterm, low birth weight, or have serious medical conditions including RD [12, 13].

Globally, there are different policies, strategies, and programs which work on or advocate for prevention and care of preterm neonates and their birth outcomes, including RD, like the Sustainable Development Goals (SDGs) and the Every Women and Every Child initiative [14, 15]. Despitethese efforts, RD remains among the leading cause of neonatal mortality and morbidity [16-20]. In Ethiopia, RD is most common cause of neonatal mortality and morbidity [16-21], resulting in exponentially increasing neonatal care costs with in the first 28 day of life. These expenditures might burden both the parents, families, and the community at large. Hence, this is the double agenda to address the survival gap of neonates which needs an inclusive investigation strategy to end the preventable cause deaths of newborns. Additionally, few studies in developing countries, including Ethiopia have provided data needed to tackle it. Therefore, in rhis study we aimed to determine the incidence and predictors of RD among neonates who were admitted to NICU at Black Lion Specialized Hospital, Ethiopia.

**Methods**

**Study design, setting, and population:** An institution-based retrospective follow-up study was conducted among a cohort of neonates from the previous consecutive five years. Our source population was all neonates admitted in NICU at Black Lion Specialized Hospital. All neonates who were admitted to NICU in the previous five consecutive years were considered as the study population.

**Eligibility criteria:** All targeted neonates’ medical cards documented in the previous five years from the study period were recruited and those with incomplete cards were excluded.

**Sample size determination and sampling procedure:** The sample size was determined via the double population proportion formula using Epi-Info Version 7 by assuming one to one ratio of exposed to non-exposed, 95% level of confidence, and power of 80%. We considered four significantly associated factors to calculate the sample size; the largest sample size was 522. After adding a 10% non-response rate, the total sample size became 604. The neonates’ cards were accessed using systematic random sampling technique after determining the sampling fraction (k=6) and the first card was selected by lottery method.

**Study variables**

**Dependent variable:** Incidence of RDS

**Independent variables:**

**Socio-demographic factors:** Neonatal-related variables included age at admission, gestational age, sex, the weight of neonate, date of NICU admission and discharge. Maternal-related variables were age and residency.

**Gynecologic-obstetric related factors:** ANC follow up, gravidity, parity, mode of delivery, multiple pregnancies, PROM, preeclampsia, abruptia placenta, and breastfeeding initiation

**Medical disorders in mother:** Hypertension, DM, HIV/AIDS

**Neonatal outcome condition:** Apgar score, RD, sepsis, jaundice, hypothermia, PNA, hypoglycemia, meningitis, esophageal atresia, CHD.

**Data collection tools:** A [retested checklist was used to collect the required data from the neonates’ charts. The checklist was translated to local language Amharic and back to English; consistency was checked. Data were collected by reviewing the complete patients' cards from the previous five consecutive years from the study period. RD was confirmed by reviewing the medical charts.

**Data quality control**: Data quality was assured by designing proper data abstraction tools. The checklist was evaluated by experienced researchers. The data collection instrument was pre-tested on 5% of the sample size. Rigorous training was given regarding the data abstraction checklist and data collection process for both data collectors and supervisors. During the data collection time, close supervision and monitoring were carried out by the supervisors and investigator. Double data entry was also done using Epi Data 4.2.0 software.

**Data processing, analysis, and presentation:** Before analysis, data was cleaned, edited, and coded. Any errors identified at this time were corrected after review of the original data using the code numbers. Data were entered using Epi-Data version 4.2.0 and analyzed using STATA 14 statistical software. Incidence density rate (IDR) was calculated for the entire study period. Subsequently, the number of cases of RD within the follow-up period was divided by the total person-time at risk on follow-up and reported per 100-person day. The Kaplan-Meier was used to estimate median mean survival time and log-rank tests were used to compare survival curves. Proportional hazard assumption was tested both graphically and through the Schoenfeld residual test for all predictors, revealing that the proportional hazard assumption is met. After checking this assumption, by comparing models, a more effective hazard model was selected using the log likelihood ratio (LR) test and the Akaike Information Criterion (AIC). In thus parametric approach, the baseline hazard and the vector of its parameters were assessed together with the regression coefficients. The best-fit model was chosen using AIC; selecting those having the smallest AIC. Then, parametric models were completed for neonates to ascertain the possible predictors. Variables having a p-value less than or equal to 0.05 in the bivariate analysis were fitted to the multivariable Gompertz hazard distribution regression model with a 95% confidence interval. A p-value less than 0.05 were considered as statistically significant.

**Operational definition**

**Event (neonatal respiratory distress):** The presence of two or more of the following signs: an abnormal respiratory rate (tachypnea>60 breaths/min, bradypnea<30 breaths/minute, respiratory pauses, or apnea) or signs of labored breathing (expiratory grunting, nasal flaring, intercostal recessions, xyphoid recessions), with or without cyanosis.

**RD:** presence of two or more of the following signs: an abnormal respiratory rate, expiratory grunting, nasal flaring, chest wall recessions, and cyanosis as per patient chart information

**Results**

**Characteristics of neonates**

Among 604 neonatal charts reviewed, 571 (94.5%) records met enrollment criteria and were included for final analysis. Of this group, 299 (52.34%) of the study participants were males. Neonates in late neonatal period accounted for more than half of the study participants. The mean age of the cohort at the time of admission to NICU was 3 days ± 3.72 SD. More than half of neonates admitted to NICU were diagnosed with neonatal sepsis, but other common causes of admissions were jaundice, hypothermia, and PNA. In addition**,** the common types of RD for neonatal admission was RDS or hyaline membrane diseases (See Figure 1)

|  | percentage |
| --- | --- |
| RDS | 43.4 |
| MAS | 34.5 |
| TTN | 15 |
| others | 7.1 |

Figure 1: Common types of RD for neonates admitted in NICU at Black Lion Specialized Hospital (n = 571).

Table 1: Characteristics of neonates admitted in NICU at Black Lion Specialized Hospital, 2018 (n = 571).

| Characteristics | Frequency | Percentage |
| --- | --- | --- |
| Sex |  |  |
| Male | 299 | 52.34 |
| Female | 272 | 47.66 |
| Maternal age |  |  |
| <20 | 61 | 10.9 |
| 20-34 | 426 | 74.6 |
| >34 | 84 | 14.7 |
| Gestational age |  |  |
| <37 | 239 | 41.8 |
| ≥37 | 332 | 58.2 |
| Neonates weight |  |  |
| <2500 | 243 | 42.6 |
| ≥2500 | 328 | 57.4 |
| Hypothermia |  |  |
| Yes | 180 | 31.5 |
| No | 391 | 68.5 |
| Sepsis | 311 | 54.5 |
| Yes | 260 | 45.5 |
| No | 247 | 43.3 |
| Jaundice |  |  |
| Yes | 202 | 35.4 |
| No | 369 | 64.6 |
| 1st minute \|APGAR |  |  |
| <7 | 337 | 59.0 |
| ≥7 | 234 | 41 |

**Socio-demographic and obstetric characteristics of mothers**

In the current study, most mothers were found to be between the ages of 20-34. The mean age of mothers was found to be 28 years ±5.42 SD . Among the total mothers enrolled into the study, 336(58.9%) had experienced spontaneous vaginal delivery and half of the neonates delivered via caesarean section developed RD during the follow-up period. Regarding obstetric, gynecological and medical diagnosis of maternal diseases, PROM 250(43.8%), HIV/AIDS (%) and DM (%) were identified. The results of this study also indicated that majority [402(70.4%)] of neonates were born to mothers who had ANC follow-up. (See Table Two).

Table 2: Socio-demographic and obstetric characteristics of mothers of neonates admitted in NICU at Black Lion Specialized Hospital from January 2018, (n = 571).

| Characteristics | Frequency | Percentage |
| --- | --- | --- |
| ANC follow-up |  |  |
| Yes | 402 | 70.4 |
| No | 169 | 29.6 |
| Maternal age |  |  |
| <20 | 61 | 10.9 |
| 20-34 | 426 | 74.6 |
| >34 | 84 | 14.7 |
| Place of delivery |  |  |
| Home | 194 | 34 |
| Health institution | 377 | 66.0 |
| Multiple pregnancy |  |  |
| Yes | 49 | 8.5 |
| No | 522 | 91.5 |
| PROM |  |  |
| Yes | 250 | 43.8 |
| No | 321 | 56.2 |
| HIV/AIDS |  |  |
| Yes | 76 | 13.3 |
| No | 495 | 86.7 |
| Maternal DM |  |  |
| Yes | 61 | 10.7 |
| No | 510 | 89.3 |

**Overall Proportion and incidence rate of RD in neonates**

This finding showed that 42.9% (95%CI: 39.3-46.1)) of neonates developed RD**.** The overall incidence of RD was also found to be 8.1 per 100 neonate day (95%CI : 7.29, 8.9) with 4331-person day observation.

**Time to discharge of neonates with RD**

The overall median length of hospital stay for neonates with RD under the study was 9 days (95%CI; 8-10) and overall length of hospital stay were 28 neonates days (IQR5, 30 neonate-days). The cumulative probability of neonates not developing RD at the end of the first day was 94.4%, at fifth to 10^th^ days was 41.3%, and at 20–28 days was 19.14% (See Figure 2).

Figure Two: Overall Kaplan-Meier survival estimate of neonates with RD admitted in NICU.

Proportional hazard assumption was evaluated using Kaplan-Meier survival and Sheffield residual global test and found to be met(x^2^ = 5.11 = P-Value =0.08) (See Figure Three).

.A B

C D

Figure 3: Kaplan-Meier survival curve of neonates with RDS admitted toNICU by **A**:PROM, **B**:PNA , **C**: Maternal HIV/AIDS and **D**:Mode of delivery.

**Model comparison criteria**

The goodness of fit model was checked using the Cox-Snell residual test. Based on the AIC, the univariate Gompertz hazard distribution (AIC = 435.8) model was more efficient than parametric exponential (AIC = 987.5) and Weibull (AIC = 686.9) semi parametric Cox-proportional hazard (AIC =1123.54) models (See Figure 4).

Figure 4: Cox-Snell residual Nelson-Aalen cumulative hazard graph on neonates with RDS admitted in NICU at Black Lion Specialized Hospital, Ethiopia, 2018 (n = 571).

**Predictors of respiratory distress**

The univariate and multivariable parametric Gompertz hazard distribution regression model was used to identify predictors of RD for neonates from admission to discharge in the NICU. Findings from bivariate analysis showed that gestational weight, being male, having no antenatal follow-up, multiple pregnancy, neonates born via caesarean section, home delivery, PROM, maternal DM, and HIV/AIDS, preterm birth, neonatal sepsis, and APGAR score less than 7 were significantly associated with time to discharge of neonates with RD. However, in the multi-variable analysis, being male, neonates born via caesarean section, home delivery, maternal DM, preterm birth, neonatal sepsis, PROM and Apgar score less than 7 were the factors which continued as statistically significant predictors of RD. The hazard of RD in male neonates was 2.4 times higher than their female counterparts [AHR: 2.4(95%CI: 1.1, 3.1)] The current study also showed that the hazard of RD among neonates born via caesarean section had nearly 2 times the increased hazard than neonates born vaginally [AHR:1,.9 (95%CI:1.6,2.3)]. In this study, the risk of RD in neonates born at home was almost 3 times higher than those delivered at health institution [AHR:2.9 (95%CI:1.5, 5,2)]. This result also indicated that neonates delivered from mothers who had DM had increased risk of RD by 2.3 times2.3(AHR:1.4 ,3.6) as compared with their non-DM counterparts [AHR 2.3(95%CI: 1.4, 3.6)]. As the gestational age increases by one week the rate of RDdecreased by 10% [AHR: 2.9(95%CI: 1.6, 5.1)]. The risk of RD was also increased by three times for a neonate who had an APGAR score less than 7 as compared with one having an APGAR score greater than or equal to 7 [AHR: 3.1 (95%CI: 1.8,5.0)] .Additionally, neonatal sepsis increases the risk of RD by 60% [AHR :1.6 (95%CI: 1.1, 2.4)]. The last predictor for RD was to be born from mothers experiencing PROM, with 1.11.1(1.8,1.5) times higher risk of RD than their counterparts not experiencing PROM [AHR: 1.1 (95%CI: 1.8, 1.5)].

**Table 3: Gompertz hazard model for predictors of incidence of RD among neonates admitted in NICU at Black Lion Specialized Hospital (N = 571)**

| Predictor | Category | RD (n, %) | Censored (n, %) | Total (n, %) | CHR (95% CI) | AHR (95% CI) |
| --- | --- | --- | --- | --- | --- | --- |
| Mother’s age | <20 | 50(20.4) | 69(21.2) | 119(10.9) | 1.5(0.97, 2.4) | 1.4(1.3,1.9) |
|  | 20-34 | 107(43.7) | 44 (13.5) | 151 (74.6) | 1 |  |
|  | ≥34 | 88(35.9) | 213(78.4) | 301 (14.7) | 2.7(1.18 ,3.4) | 2.8(1.8,3.3) |
| Sex | Female | 171(48.6) | 101(46.2.) | 272(47.6) | 1 |  |
|  | Male | 181(51.4) | 118 (53.8) | 299(52.4) | 1.7(1.2 , 2.3)** | 2.4(1.1,3.1) * |
| Place of delivery | Home |  |  |  | 3.14 (2.3, 5.2) | 2.9 (1.5, 5,2)* |
|  | Health institution |  |  |  | 1 |  |
| ANC follow up | Yes | 56(22.8) | 113 (34.7) | 169 (29.6) | 0.4(0.3,0.5) ** | 0.8(0.54, 1.19) |
|  | No | 189(77.2) | 213(65.3) | 402(70.4) | 1 |  |
| Multiple pregnancy | Yes | 23 (9.4) | 26(8) | 49(8.5) | 1.6(1.1 ,2.1)** | 1.1(0.9,1.6) |
|  | No | 22(90.6) | 300(92) | 351(91.5) | 1 |  |
| PROM | Yes | 143(58.4) | 107(32.8) | 250(43.8) | 1.5(1.1, 2.0)* | 1.1(1.8,1.5)* |
|  | No | 102(41.6) | 219(67.2) | 321(56.2) | 1 |  |
| Mode of delivery | Caesarean section | 132(53.8) | 103(31.6) | 235(41.2) | 1.6(1.2, 2.2)** | 1.9(1.6,2.3) |
|  | Vaginal Caesarean section | 113(46.2) | 223(68.4) | 336(58.8) | 1 |  |
| HIV/AIDS | Yes | 35(14.3) | 41(12.6) | 76(13.3) | 1.9(1.3,2.7)** | 1.5(0.9, 2.5) |
|  | No | 210(85.7) | 285 (87.4) | 495(86.7) | 1 |  |
| Maternal DM | Yes | 39(15.9) | 22(6.7) | 61(10.7) | 2.4(1.6,3.5)** | 2.3(1.4 ,3.6)** |
|  | No | 206(84.1) | 304 (93.3) | 510(89.3) | 1 |  |
| Sepsis | Yes | 189(77.1) | 122(37.4) | 311 (54.5) | 2.2(1.6,3.1)** | 1.6(1.1, 2.4)** |
|  | No | 56(22.9) | 204(62.6) | 260(45.5) | 1 |  |
| GA | <37 | 23(13.5) | 8 | 31(5.4) | 6.3(3.9 ,10.2)** | 2.9(1.6, 5.1)** |
|  | ≥37 | 61(35.9) | 271(67.6) | 332(58.1) | 1 |  |
| Neonatal Wight | <1000 | 22 (12.9) | 11 | 33 (5.8) | 3.8(1.9, 7.5)** | 1.9(0.9,4.3) |
|  | 1000-1500 | 56(32.9) | 99(24.7) | 155 ( 27.1) | 1.3 (0.7, 2.4) | 0.8(0.41, 1.6) |
|  | 1500-2500 | 84(49.4) | 257(64.1) | 341 (59.7) | 1.1 (0.6, 1.9) | 0.8(0.4, 1.4) |
|  | ≥2500 | 8 | 34(8.5) | 42( 7.4) | 1 |  |
| First minute APGAR  Score | <7 | 154(90.6) | 283 (70.5) | 437 (76.5) | 3.2(1.9,5.4)* | 3.1 (1.8,5.0)* |
|  | ≥7 | 16 | 118 (29.5) | 134 (23.5) | 1 |  |
| Fifth minute APGAR score | <7 | 128(75.2) | 131(32.7) | 259(45.4) | 3.8(2.7, 5.4)** | 1.81(1.3,4.8)** |
|  | ≥7 | 42(24.8) | 270(67.3) | 312 (54.6) | 1 |  |

**Discussion**

In the current study, the overall proportion of RD among neonates admitted in Black Lion Specialized Hospital NICU was 42.9% (95%CI: 39.3-46.1%). This finding is in line with a study reported in the Republic of China [22]. However, our finding is higher than a study conducted in several countries including Nepal (34%) [23], India (33.4%) [9], Egypt (34.3%) [24], Pakistan (4.6%) [25], Northern Italy (20.1%) [26], and Portugal (8.83%) [27]. Such variance might be due to the difference in the study setting which may be more advanced in terms of maternal newborn care services. Additionally, sample size, study design, and population socio-demographic characteristics may yield difference in the findings. Our finding was lower than studies from Saudi Arabia (54.7%) [7], Cameroon (47.5%) [6] and Poland (54.29%) [28]. This difference may reflect quality/qualifications of staff, public awareness to attended births, and cultural beliefs.

Based on the current finding, the overall incidence of RD was 8.1 per 100 neonate-days (95%CI: 7.3, 8.9). The common causes of respiratory distress in our study were RDS and meconium aspiration, which is similar to previous findings from Nepal and Egypt [23, 24].

The predictors of RD were complex and multiple. Preterm birth, caesarean section delivery, Apgar score < 7, sepsis, PROM, maternal DM, and home delivery were found to be predictors of RD. The risk of RD in male neonates was 2.4 times higher than their female counterparts; a finding which is similarly reflect in studies done in China [27] and Cameroon [6]. This aligns with scientific knowledge that male neonates have higher levels of circulating testosterone than females which may be associated with differences in pulmonary biomechanics and vascular development among males.

For those neonates delivered via caesarean section there was nearly 2 times more likelihood of RD risk than non-caesarean births – a finding similarly reported China [22], Cameroon [6], and Italy [29].

In this study, the risk of RD for neonates born at home was almost 3 times higher than those delivered at health institution.

Mothers with DM bore infants 2.3 times more likely to develop RD than non-DM mothers. 2; again a finding supported by research done in China [30]. It is possible this relates to the fact that these neonates have plentiful glucose stores , but develop hypoglycemia because of high insulin secretion induced by maternal and fetal hyperglycemia.

Our study presented found a threefold greater likelihood of RD amongst preterm neonates than those who were term births, which align with work from Cameron [6] and Italy [29]. This finding seems to align with the positive association between gestational age and fetal development, thereby reducing complications as the level of prematurity is lessened. Additionally, those neonates born as preterm often have immature lung structures which might delay intrapulmonary fluid absorption, surfactant deficiency, and inefficient gas exchange. The risk of RD was also increased by three times for a neonate who had an APGAR score less than 7, which has been previously reported in other studies [6, 25, 26]. Finally, neonatal sepsis was significantly associated with the risk of developing RD as was found in in Nepal and Egypt, [23, 24].

**Conclusion** The incidence of RD among neonates at the Black Lion Specialized Hospital in Addis Ababa, Ethiopia was found to be high. Those neonates delivered at home, delivered through caesarean section, preterm neonates, whose APGAR score<7, and born from diabetic mothers were most likely to develop RD. Hence, it is indicated to promote health institutional delivery, especially in instances of premature labour/birth possibilities.

**Abbreviations**

APGAR: Appearance pulse grimace activity respiration; CI: Confidence interval; COR: Crude odd ratio; GA: Gestational age; HIV: Human immunodeficiency virus; HMD: Hyaline membrane disease; HR: Hazard ratio; NICU: Neonatal intensive care unit; PNA: Perinatal asphyxia; PROM: Prolonged rupture of membrane; RD: Respiratory distress. MAS, TTN

**Declarations**

**Ethical consideration:** Ethical clearance was obtained from Addis Ababa University, College of Nursing and Midwifery Research Committee. Letters of cooperation were written to Black Lion Specialized Hospital and subsequently permission was obtained from the clinical director and relevant department and unit heads of the hospital. Following these approvals, access to the medical charts was undertaken with due diligence to maintain participant confidentiality.

**Consent for publication:** The study was not a clinical trial that needs direct involvement of the patient and did not elicit an injury to participants since the data is retrospectively collected from the patient chart. So, there was no need of consent for publication.

**Data Availability statement:** The data used in this manuscript can be easily availed with email contact of the main authors. Data made available to all interested researchers upon request.

**Competing interest:** The authors declare that they have no conflicts of interest.

**Funding**: The authors have also confirmed that no financial funding in the study, authorship and publication of this article was received.

**Authors’ contributions:** All authors contributed to the design of the study and the interpretation of data. YA performed the data analysis and compiled the work. YA and HM drafted the manuscript. All authors critically revised the draft manuscript. All authors read and approved the final manuscript.

**References**

1.	Hooper SB, Te Pas AB, Kitchen MJ. **Respiratory transition in the newborn: a three-phase process.** Archives of Diseases Child Fetal Neonatal Ed 2016; 101:F266.

2.	WHO, UNICEF, World Bank. **State of the world’s vaccines and immunization, 3rd ed.** Geneva, World Health Organization, 2009.

3.	Edwards MO, Kotecha SJ, Kotecha S. **Respiratory distress of the term newborn infant.** Paediatric Respiratory Review. 2013;14(1):29–36.

4.	Hibbard JU, Wilkins I, Sun L, et al.**: Consortium on Safe Labor. Respiratory morbidity in late preterm births.** Journal of the American Medical Association. 2010;304(4):419–425.

5.	Assefa NE, Berhe H, Girma F, Berhe K, Berhe YZ, Gebrehet G, Werid WM, Berhe A, Rufae HB, Welu G: **Risk factors of premature rupture of membranes in public hospitals at Mekele city, Tigray, a case control study**. BMC pregnancy and childbirth 2018, **18**(1):386.

6.	Tochie JN, Choukem S-P, Langmia RN, Barla E, Koki-Ndombo P: **Neonatal respiratory distress in a reference neonatal unit in Cameroon: an analysis of prevalence, predictors, etiologies and outcomes**. Pan African Medical Journal 2016, **24**(1).

7.	Qari SA, Alsufyani AA, Muathin SH, El Margoushy NM: **Prevalence of Respiratory Distress Syndrome in Neonates**. Egyptian Journal of Hospital Medicine 2018, **70**(1).

8.	Sathenahalli V, Dwivedi D, Bajaj N, Singh H: **Predictors of poor outcome in neonates with respiratory distress**. Int J Contemp Pediatr 2016, **3**:76-79.

9.	Singh S, Srivastava R, Singh A, Tahazzul M, Kumar M, Kanta C, Chandra S: **Respiratory distress including meconium aspiration syndrome in vigorous neonates born through meconium stained amniotic fluid: incidence, onset, severity and predictors at birth**. The Indian Journal of Pediatrics 2013, 80(7):538-543.

10.	Hermansen CL, Lorah KN: **Respiratory distress in the newborn**. American Family Physician 2007, **76**(7):987-994.

11.	Edwards MO, Kotecha SJ, Kotecha S: **Respiratory distress of the term newborn infant**. Paediatric respiratory reviews 2013, **14**(1):29-37.

12.	Wayne.daniel.biostatics a Foundation for Analysis in the Health Sciences.13th.ed.Georgia State University; ©2013.chapter 6 dossfepp-.

13.	**United Nations Inter-agency Group for Child Mortality Estimation (UN IGME), ‘Levels & Trends in Child Mortality: Report 2017, Estimates Developed by the UN Inter-agency Group for Child Mortality Estimation’, United Nations Children’s Fund, New York, 2017.**

14.	United Nations: **Transforming our world: The 2030 Agenda for Sustainable Development,**. 2015.

15.	Child. EWE: **THE GLOBAL STRATEGY FOR WOMEN’S, CHILDREN’S AND ADOLESCENTS’ HEALTH (2016-2030):**. In. Geneva; 2015.

16.	WHO.: **Born Too Soon The Global Action Report on PretermBirth.** In. Geneva; 2012: 128.

17.	March of Dimes WHO: **The Global Action Report on Preterm Birth**. Geneva: WHO; 2016.

18.	Kliegman RIN, Waldo E. (Waldo Emerson): **Nelson Textbook Of Pediatrics. Chapter 91: Prematurity and Interauterine restriction** In., edn. United states of America: Elsevier; 2011.

19.	Joy E Lawn MGG, Toni M Nunes, Craig E Rubens, Cynthia Stanton, and the GAPPS Review Group: **Global report on preterm birth and stillbirth (1 of 7): definitions, description of the burden and opportunities to improve data**. BMC Pregnancy and Childbirth 2010, **10**:22.

20.	Mengesha HG, Sahle BW: **Cause of neonatal deaths in Northern Ethiopia: a prospective cohort study**. BMC Public Health 2017, **17**(1):62.

21.	FMOH: **Health and Health Related Indicators**. In., vol. 2. Ethiopia: FMOH; 2013: 68.

22.	Li Y, Zhang C, Zhang D: **Cesarean section and the risk of neonatal respiratory distress syndrome: a meta-analysis**. Archives of Gynecology and Obstetrics 2019:1-15.

23.	Rijal P, Shrestha M: **Scenario of Neonatal Respiratory Distress in Tertiary Hospital**. Birth 2018, **14**(10):6.

24.	Laban M, Mansour GM, Elsafty MS, Hassanin AS, EzzElarab SS: **Prediction of neonatal respiratory distress syndrome in term pregnancies by assessment of fetal lung volume and pulmonary artery resistance index**. International Journal of Gynecology & Obstetrics 2015, **128**(3):246-250.

25.	Parkash A, Haider N, Khoso ZA, Shaikh AS: **Frequency, causes and outcome of neonates with respiratory distress admitted to Neonatal Intensive Care Unit, National Institute of Child Health, Karachi**. Journal of Pakistan Medical Association 2015, **65**(7):771-775.

26.	Bonet‐Carne E, Palacio M, Cobo T, Perez‐Moreno A, Lopez M, Piraquive J, Ramirez J, Botet F, Marques F, Gratacos E: **Quantitative ultrasound texture analysis of fetal lungs to predict neonatal respiratory morbidity**. Ultrasound in Obstetrics & Gynecology 2015, **45**(4):427-433.

27.	Correia C, Rocha G, Flor-de-Lima F, Guimarães H: **Respiratory morbidity in late preterm infants**. Minerva Pediatrica 2018, **70**(4):345-354.

28.	Niesłuchowska-Hoxha A, Cnota W, Czuba B, Ruci A, Ciaciura-Jarno M, Jagielska A, Wójtowicz D, Kierach R, Dąbrowski K, Sidorowicz M: **A Retrospective Study on the Risk of Respiratory Distress Syndrome in Singleton Pregnancies with Preterm Premature Rupture of Membranes between 24**. BioMed Research International 2018, **2018**.

29.	Condò V, Cipriani S, Colnaghi M, Bellù R, Zanini R, Bulfoni C, Parazzini F, Mosca F: **Neonatal respiratory distress syndrome: are risk factors the same in preterm and term infants?** The Journal of Maternal-Fetal & Neonatal Medicine 2017, **30**(11):1267-1272.

30.	Li Y, Wang W, Zhang D: **Maternal diabetes mellitus and risk of neonatal respiratory distress syndrome: a meta-analysis**. Acta Diabetologica 2019:1-12.
