# Supplementary material for: Incidence of respiratory distress and its predictors among neonates admitted to the neonatal intensive care unit, Black Lion Specialized Hospital, Addis Ababa, Ethiopia
Source: PLoS One. 2020 Jul 1;15(7):e0235544. doi: 10.1371/journal.pone.0235544 (PMC7329073; doi:10.1371/journal.pone.0235544)
Supplement: Attachment — Submitted filename: Response to editor.docx [file pone.0235544.s005.docx]

**Dear Academic Editor!**

**PLOS ONE**

**Response to Academic Editor**

I am pleased to resubmit for publication version of “***Incidence of respiratory distress and its predictors among neonates admitted to the neonatal intensive care unit, Black Lion Specialized Hospital, Addis Ababa, Ethiopia*.**” for a review as original research in **International Journal of Pediatrics**.

The comments of the editor and the reviewers were highly insightful and enabled us to greatly improve the quality of our manuscript. Therefore, based on the editor’s concerns we have made extensive edition in our manuscript.

In the following pages, we have addressed yours’ concerns in a point by point format.

We look forward to hearing from you at your earliest convenience.

Thank you for your consideration of this manuscript!

Kind regards,

**Yared Asmare Aynalem.**

On behalf of authors

**Editors comment**

**PLOS ONE**

PONE-D-20-01297R1
Incidence of respiratory distress and its predictors among neonates admitted to the neonatal intensive care unit, Black Lion Specialized Hospital, Addis Ababa, Ethiopia
mr Yared Asmare Aynalem

Dear mr Aynalem,

Thank you for submitting your manuscript entitled "Incidence of respiratory distress and its predictors among neonates admitted to the neonatal intensive care unit, Black Lion Specialized Hospital, Addis Ababa, Ethiopia" to PLOS ONE.  Your manuscript files have been checked in-house but before we can proceed we need you to address the following issues:

Response: thank you for contacting as

1. We note that your author list was updated during the revision process. Please complete our Authorship Change Form by following this link: <http://journals.plos.org/plosone/s/file?id=13d0/plos-one-change-to-authorship-form.docx>. You may return the form as an attachment by emailing [plosone@plos.org](mailto:plosone@plos.org) or by uploading it as a submission file labeled with the file type ‘Other’. Please note that if your manuscript is accepted, we will not be able to complete the publication process without the completed form.

Response: thank you for reminding as to complete our Authorship Change Form. We have complet it and we sent to [plosone@plos.org](mailto:plosone@plos.org) 

2. Please include a separate legend for each figure in your manuscript.
Response: thank you. We have included a separate legend for each figure in your manuscript.
3. Please ensure that you refer to Table 1 and 3 in your text as, if accepted, production will need this reference to link the reader to the Tables.
Response: thank you. We have cite Table 1 and 3 in the text
4. Thank you for updating your data availability statement. You note that your data are available within the Supporting Information files, but no such files have been included with your submission. At this time we ask that you please upload your minimal data set as a Supporting Information file, or to a public repository such as Figshare or Dryad.

Response: thank you. The only data that were not send to the journal is the checklist that we have used to collect the data.so that we have attached it as Supporting Information files
Please also ensure that when you upload your file you include separate captions for your supplementary files at the end of your manuscript.

Response: thank you. We have included a separate captions for our supplementary files at the end of your manuscript.

As soon as you confirm the location of the data underlying your findings, we will be able to proceed with the review of your submission.

Response: thank you.

Your manuscript has been returned to your account. Please log on to PLOS Editorial Manager at <https://www.editorialmanager.com/pone/> to access your manuscript.

Your manuscript can be found in the "Revisions Sent Back to the Author" link under the New Submissions menu. After you have made the changes requested above, please be sure to view and approve the revised PDF after rebuilding the PDF to complete the resubmission process.

Please note that these changes have been requested to comply with submission guidelines and your manuscript will *not* be sent to review until you have fully adhered to our requests.  Once your paper has been seen by an Editor we may return it to you for further information or amendments.

We ask that you address this request within 28 days. If you require additional time, please email the journal office. We are happy to grant extensions of up to one month past this due date. If we have not heard from you within 28 days, your manuscript will be withdrawn from Editorial Manager.

Thank you for submitting your work to PLOS ONE.

Kind regards,

Jo George
PLOS ONE
