# Supplementary material for: Incidence of respiratory distress and its predictors among neonates admitted to the neonatal intensive care unit, Black Lion Specialized Hospital, Addis Ababa, Ethiopia
Source: PLoS One. 2020 Jul 1;15(7):e0235544. doi: 10.1371/journal.pone.0235544 (PMC7329073; doi:10.1371/journal.pone.0235544)
Supplement: Attachment — Submitted filename: Response to Reviewers.docx [file pone.0235544.s006.docx]

**Dear Academic Editor!**

**PLOS ONE**

**A letter Accompanying Revision in Response to editor and Reviewers Comment**

I am pleased to resubmit for publication version of “**Incidence of respiratory distress and its predictors among neonates admitted to the neonatal intensive care unit, Black Lion Specialized Hospital, Addis Ababa, Ethiopia”** for a review as original research in **PLOS ONE.**

financial disclosure: The authors confirmed that no financial funding was received for the study. None of the authors received specific funding for this work.

The comments of the editor and reviewers were highly insightful and enabled us to greatly improve the quality of our manuscript. Therefore, based on the editor and reviewers’ concerns we have made extensive edition in our manuscript. In the following pages, we have addressed yours’ concerns in a point by point format.

We look forward to hearing from you at your earliest convenience.

Thank you for your consideration of this manuscript!

Sincerely,

**Yared Asmare Aynalem**

On behalf of authors

PONE-D-20-01297R1
Incidence of respiratory distress and its predictors among neonates admitted to the neonatal intensive care unit, Black Lion Specialized Hospital, Addis Ababa, Ethiopia
PLOS ONE

Dear Dr. Yared Asmare Aynalem,

Thank you for submitting your manuscript to PLOS ONE. After careful consideration, we feel that it has merit but does not fully meet PLOS ONE’s publication criteria as it currently stands. Therefore, we invite you to submit a revised version of the manuscript that addresses the points raised during the review process.
Response: We thank you very much for consideration
Please submit your revised manuscript by July 31 2020. If you will need more time than this to complete your revisions, please reply to this message or contact the journal office at [plosone@plos.org](mailto:plosone@plos.org). When you're ready to submit your revision, log on to <https://www.editorialmanager.com/pone/> and select the 'Submissions Needing Revision' folder to locate your manuscript file.
Response: We thank you very much. We have submitted it.

Please include the following items when submitting your revised manuscript:

- A rebuttal letter that responds to each point raised by the academic editor and reviewer(s). You should upload this letter as a separate file labeled 'Response to Reviewers'.
- A marked-up copy of your manuscript that highlights changes made to the original version. You should upload this as a separate file labeled 'Revised Manuscript with Track Changes'.
- An unmarked version of your revised paper without tracked changes. You should upload this as a separate file labeled 'Manuscript'.

Response: We thank you very much. We have submitted Response to Reviewers, Revised Manuscript with Track Changes and Manuscript separately

If you would like to make changes to your financial disclosure, please include your updated statement in your cover letter. Guidelines for resubmitting your figure files are available below the reviewer comments at the end of this letter.

Response: We thank you. We have included the financial disclosure as The authors have also confirmed that no financial funding was received for the study, authorship, and publication of this article.
the figures were uploaded with the plos one guide line
If applicable, we recommend that you deposit your laboratory protocols in [protocols.io](http://protocols.io/) to enhance the reproducibility of your results. Protocols.io assigns your protocol its own identifier (DOI) so that it can be cited independently in the future. For instructions see: <http://journals.plos.org/plosone/s/submission-guidelines#loc-laboratory-protocols>

Response. Thank you. We think that it is not applicable

We look forward to receiving your revised manuscript.

Kind regards,

Georg M. Schmölzer
Academic Editor
PLOS ONE

Response. Big thanks. We will send it as soon as possible
Response
**Reviewer #**
Reviewers' comments:

Reviewer's Responses to Questions

**Comments to the Author**

1. If the authors have adequately addressed your comments raised in a previous round of review and you feel that this manuscript is now acceptable for publication, you may indicate that here to bypass the “Comments to the Author” section, enter your conflict of interest statement in the “Confidential to Editor” section, and submit your "Accept" recommendation.

Reviewer #1: All comments have been addressed

Response: thank you very much for acknowledging it

2. Is the manuscript technically sound, and do the data support the conclusions?

The manuscript must describe a technically sound piece of scientific research with data that supports the conclusions. Experiments must have been conducted rigorously, with appropriate controls, replication, and sample sizes. The conclusions must be drawn appropriately based on the data presented.

Reviewer #1: Yes

 Response: thank you very much.

3. Has the statistical analysis been performed appropriately and rigorously?

Reviewer #1: Yes

 Response: thank you very much.

4. Have the authors made all data underlying the findings in their manuscript fully available?

The [PLOS Data policy](http://www.plosone.org/static/policies.action#sharing) requires authors to make all data underlying the findings described in their manuscript fully available without restriction, with rare exception (please refer to the Data Availability Statement in the manuscript PDF file). The data should be provided as part of the manuscript or its supporting information, or deposited to a public repository. For example, in addition to summary statistics, the data points behind means, medians and variance measures should be available. If there are restrictions on publicly sharing data—e.g. participant privacy or use of data from a third party—those must be specified.

Reviewer #1: Yes

 Response: thank you very much.

5. Is the manuscript presented in an intelligible fashion and written in standard English?

PLOS ONE does not copyedit accepted manuscripts, so the language in submitted articles must be clear, correct, and unambiguous. Any typographical or grammatical errors should be corrected at revision, so please note any specific errors here.

Reviewer #1: Yes

 Response: thank you very much.

6. Review Comments to the Author

Please use the space provided to explain your answers to the questions above. You may also include additional comments for the author, including concerns about dual publication, research ethics, or publication ethics. (Please upload your review as an attachment if it exceeds 20,000 characters)

Reviewer #1: Under Background
Probably more correct to say. “It is often seen during the transition from fetal to neonatal life”. Not triggered by

Response: thank you very much. **Response: Based on your recommendations, we have addressed the issue. See the track change**

7. PLOS authors have the option to publish the peer review history of their article ([what does this mean?](https://journals.plos.org/plosone/s/editorial-and-peer-review-process#loc-peer-review-history)). If published, this will include your full peer review and any attached files.


If you choose “no”, your identity will remain anonymous but your review may still be made public.


**Do you want your identity to be public for this peer review?** For information about this choice, including consent withdrawal, please see our [Privacy Policy](https://www.plos.org/privacy-policy).

Reviewer #1: No
**Response: Thank you**


While revising your submission, please upload your figure files to the Preflight Analysis and Conversion Engine (PACE) digital diagnostic tool, <https://pacev2.apexcovantage.com/>. PACE helps ensure that figures meet PLOS requirements. To use PACE, you must first register as a user. Registration is free. Then, login and navigate to the UPLOAD tab, where you will find detailed instructions on how to use the tool. If you encounter any issues or have any questions when using PACE, please email PLOS at [figures@plos.org](mailto:figures@plos.org). Please note that Supporting Information files do not need this step.

**Response: thank you. We have** uploaded figure files to the Preflight Analysis and Conversion Engine (PACE) digital diagnostic tool.
